# Ammonium and organic carbon co-removal under feammox-coupled-with-heterotrophy condition as an efficient approach for nitrogen treatment

**DOI:** 10.1038/s41598-020-80057-y

**Published:** 2021-01-12

**Authors:** Chung Phuong Le, Hai Thi Nguyen, Toi Duy Nguyen, Quyen Huynh Minh Nguyen, Hai The Pham, Hang Thuy Dinh

**Affiliations:** 1 Nha Trang University, Nguyen Dinh Chieu 2, Nhatrang, Khanhhoa Vietnam; 2 VNU Institute of Microbiology and Biotechnology, E2 Building, Xuan Thuy 144, Caugiay, Hanoi, Vietnam; 3 GREENLAB-Center for Life Science Research (CELIFE), VNU University of Science, Nguyen Trai 334, Thanh Xuan, Hanoi, Vietnam; 4 Department of Microbiology, Faculty of Biology, VNU University of Science, Nguyen Trai 334, Thanh Xuan, Hanoi, Vietnam

**Keywords:** Microbiology, Environmental sciences

## Abstract

Nitrification is the rate limiting step in the nitrogen removal processes since nitrifiers have high oxygen demand, but poorly compete with aerobic heterotrophs. In a laboratory-scaled system, we investigated a process of ammonium oxidation under ferric-iron reducing condition (feammox) in the presence of organic carbon using influents with high NH_4_^+^ and COD contents, and ferrihydrite as the only electron acceptor. Batch incubations testing influents with different NH_4_^+^ and COD concentrations revealed that the [COD]/[NH_4_^+^] ratio of 1.4 and the influent redox potential ranging from − 20 to + 20 mV led to the highest removal efficiencies, i.e. 98.3% for NH_4_^+^ and 58.8% for COD. N_2_ was detected as the only product of NH_4_^+^ conversion, whereas NO_2_^−^ and NO_3_^−^ were not detected. While operating continuously with influent having a [COD]/[NH_4_^+^] ratio of 1.4, the system efficiently removed NH_4_^+^ (> 91%) and COD (> 54%) within 6 day retention time. Fluorescence in situ hybridization analyses using Cy3-labeled 16S rRNA oligonucleotide probes revealed that gamma-proteobacteria dominated in the microbial community attaching to the matrix bed of the system. The iron-reduction dependent NH_4_^+^ and COD co-removal with a thorough conversion of NH_4_^+^ to N_2_ demonstrated in this study would be a novel approach for nitrogen treatment.

## Introduction

Wastewaters with high ammonia content if discharged inappropriately can cause adverse environmental effects to aquatic systems, i.e. toxicity to living organisms and eutrophication in water bodies^1^. Ammonium removal from wastewater has been for long time a controversial research topic for the scientific community. Traditionally, NH_4_^+^ is removed by the two-stage process of nitrification (aerobic, lithotrophic) and denitrification (anaerobic, organotrophic)^2^. An alternative technology for NH_4_^+^ removal is the partial nitritation/anammox process in which half NH_4_^+^ is first oxidized to nitrite by nitrifying bacteria, afterward the other half is oxidized with nitrite to N_2_ by *Planctomycetes* species^3^. Both technologies employ nitrification step which is highly demanding in oxygen and chiefly responsible for the elevated cost of the treatment process. Although the partial nitritation/anammox reduces up to 60% of that cost, technologies with no or lower oxygen demand are still desired.

In the last decade, a novel pathway of NH_4_^+^ oxidation coupled to ferric iron (Fe^3+^) reduction in the absence of oxygen and mediated by microorganisms, termed feammox was proposed^4–7^. Clement et al.^8^ and Shrestha et al.^5^ reported the feammox process in wetland soils, where NH_4_^+^ was oxidized to NO_2_^*−*^ and considered as a significant mechanism of nitrogen loss in wetland environment^5,8^. In a laboratory model, Sawayama^4^ demonstrated the feammox process with NH_4_^+^ conversion to NO_2_^−^, and thus proposed feammox as an alternative mode of NH_4_^+^ removal from wastewater. Using the 16S rRNA gene clone library approach, the major microbial groups in the model were identified as *Exiguobacterium* spp., *Pseudomonas* spp. and *Carnobacterium maltaromaticum*^4^. None of the delta-proteobacterial iron reducers was detected.

In other studies, NH_4_^+^ oxidation directly to N_2_ was also proven for microbial communities in tropical rainforest soil^6^. The first pure culture of feammox bacterium was strain A6 of the *Acidimicrobiaceae* group (supposed to be a new taxon) isolated from a low pH ferrihydrite-containing enrichment culture. This strain grew under autotrophic and acidic condition (at pH 5) and used ferric iron to oxidize NH_4_^+^ to nitrite^9^. With the ability of oxidizing NH_4_^+^ via the feammox pathway, strain A6 was proposed for application in a new feammox-based process of NH_4_^+^ removal from wastewater^10^.

In the feammox process, NH_4_^+^ can be oxidized to NO_2_^–^, NO_3_^–^ or N_2_, depending on the environmental pH, and thus the amount of energy retrieved by microorganisms can be respectively at different levels^4,5,8,11^:

1 $${\text{3Fe}}\left( {{\text{OH}}} \right)_{{3}} + {\text{ 5H}}^{ + } + {\text{ NH}}_{{4}}{^{ + }} \to {\text{ 3Fe}}^{{{2} + }} + {\text{ 9H}}_{{2}} {\text{O }} + \, 0.{\text{5N}}_{{2}} \quad \left( { - {\text{245 kJ mol}}^{{ - {1}}} } \right)$$

2 $${\text{6Fe}}\left( {{\text{OH}}} \right)_{{3}} + { 1}0{\text{H}}^{ + } + {\text{ NH}}_{{4}}{^{ + }} \to {\text{ 6Fe}}^{{{2} + }} + {\text{ 16H}}_{{2}} {\text{O }} + {\text{ NO}}_{{2}}^{-} \quad \left( { - {\text{164 kJ mol}}^{{ - {1}}} } \right)$$

3 $${\text{6Fe}}\left( {{\text{OH}}} \right)_{{3}} + {\text{ 8H}}^{ + } + {\text{ NH}}_{{4}}{^{ + }} \to {\text{ 6Fe}}^{{{2} + }} + {\text{ 15H}}_{{2}} {\text{O }} + {\text{ NO}}_{{3}}^{-} \quad \left( { - {2}0{\text{7 kJ mol}}^{{ - {1}}} } \right)$$

The conversion of NH_4_^+^ directly to N_2_ (Eq. 1) is energetically most favorable and likely to occur in a wide range of environmental pH conditions, whereas NH_4_^+^ conversion to NO_2_^–^ or NO_3_^–^ (Eqs. 2 and 3) are more likely to occur in acidic environments (pH < 6.5)^6^.

Generally, in the practice of wastewater treatment, COD if presented at high concentrations is needed to be removed prior to NH_4_^+^ removal step (via anaerobic technologies such as biogas reactor, upflow anaerobic sludge blanket—UASB), because nitrifying microorganisms are litho-autotrophic, highly require oxygen for the ammonium oxidation, although they poorly compete with heterotrophic species for oxygen in environments where organic carbon sources are available^12^.

In this paper, we report a process of NH_4_^+^ oxidation in the presence of organic carbon in a laboratory-scaled anaerobic system with ferrihydrite as the only electron acceptor (heterotrophic feammox). The system could achieve a thorough conversion of NH_4_^+^ to N_2_ while being operated under neutral pH conditions, i.e. close to the conditions of most NH_4_^+^ and COD bearing wastewater sources in daily life. The co-removal of NH_4_^+^ and COD coupled with iron reduction demonstrated in this study provides an alternative nitrogen treatment technology that does not require oxygen, thereby eliminating the most critical disadvantage of the presently applied technologies based on nitrification/denitrification and partial nitritation/anammox principles.

## Materials and methods

### Laboratory-scaled treatment system design

**Figure 1:**
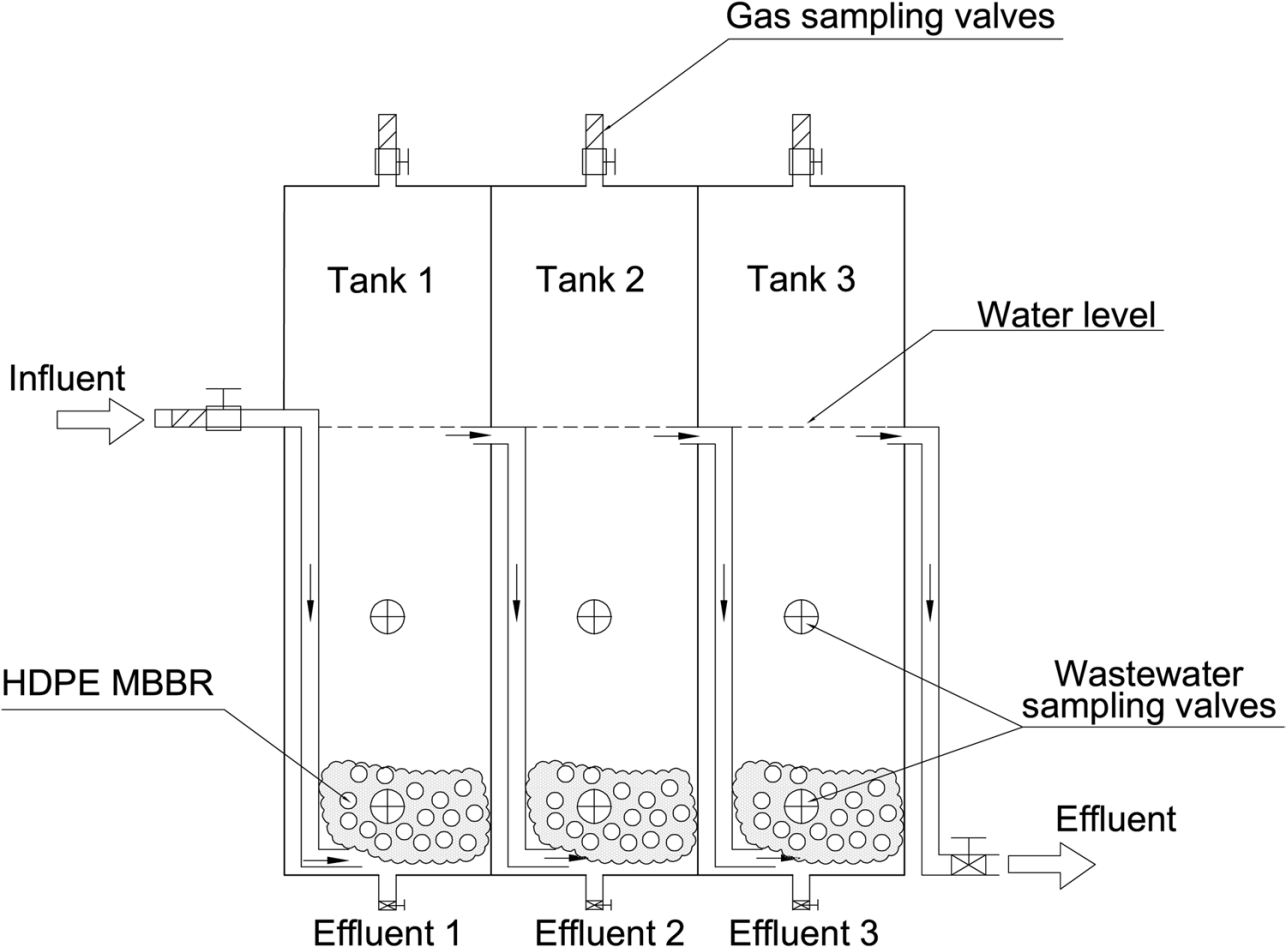
Schematic diagram of the laboratory-scaled treatment system. In batch experiments, three tanks of the system operated independently with influents of different compositions. In continuous operation, the three tanks worked as a unique system in which the influent flowed from one tank to the next one via bottom-oriented connecting tubes.

The laboratory-scaled treatment system in this study was designed following the working principle of a wastewater treatment plant (Fig. S1) in Cu Chi (HCM City, Vietnam) that operates in the presence of Fe-containing anaerobic sludge. The laboratory system consisted of three connected acrylic tanks of 20 cm × 12 cm × 40 cm (length × width × height) size. The working volume of each tank was 6 L, and the total working volume of the system was 18 L. Liquid medium as the influent was supplied by a peristaltic pump, and subsequently transferred from one tank to the next by overflow through bottom-oriented connecting tubes to ensure good contact of the influent with the microbial community at the bottom of each tank. A matrix bed consisting of 100 cm^3^ of HDPE MBBR matrix 12 × 7 mm (specific surface 850 m^2^/m^3^, Kaite Chemical, PRC) was loaded onto every tank. Each tank has a gas sampling valve positioned on the lid, and two water sampling valves positioned at low and mid-height levels (at 3.5 cm and 13.5 cm from the bottom, respectively, while the full water level was 27 cm) (Fig. 1).

The mineral medium that was used as the influent was adapted from Ratering and contained the following components^13^: 1.0 g NaCl L^−1^, 0.4 g MgCl_2_·6H_2_O L^−1^, 0.15 CaCl_2_·2H_2_O L^−1^, 0.5 KCl L^−1^, 0.2 KH_2_PO_4_ L^−1^ (pH 7 ± 0.2). Ammonium and COD were loaded at different concentrations depending on the experimental conditions by using solutions of 1.0 mM NH_4_Cl and 1.0 M sodium acetate, respectively. The influent was then supplemented with 1 mL L^−1^ vitamin and microelement solutions^14^. Afterward, the buffering bicarbonate and reducing agent sodium ascorbate solutions were added to the final concentrations of 30 mM and 1 mM, respectively. The slurry of the electron acceptor ferrihydrite was freshly prepared according to Ratering and added to the medium for a final concentration of 30 mM^13^. Finally, the synthetic wastewater was flushed with argon to remove dissolved oxygen.

### Enrichment of iron-reducing community in the laboratory-scaled system

Enrichment of iron-reducing microorganisms in the laboratory-scaled system was carried out by circulating the anoxic influent inside the system (from the first tank to the third tank and back) with a peristaltic pump (Huiyu Weiye (Beijing), PRC) at 2 mL min^−1^. The inoculum was a Fe-containing sludge from the wastewater treatment plant in Cu Chi, HCM City Vietnam. Accordingly, 1.5 kg seed sludge was resuspended in 3 L anoxic distilled water in a tightly sealed glass bottle, shaken at 100 rpm for 5 min, then added to each tank in a ratio of 10% of the working volume. The tanks were flushed with argon to remove oxygen and then sealed with gas-tight lids. The enrichment process was carried out at ambient temperatures (30 ± 2 °C). Gas samples from the head space of each tank were collected every 24 h for N_2_ analyses. In parallel, water samples from each tank were collected every 24 h for analyses of the relevant parameters, including NH_4_^+^, COD, and Fe^2+^. A negative control was established in a separate tank containing mineral medium with compositions similar to those of the medium in the enrichment tanks, except that the organic carbon source (as sodium acetate) was omitted.

### Batch incubation experiment

After enrichment, the influent of the system was replaced with a freshly prepared anoxic medium containing 200 mg L^−1^ NH_4_^+^ and COD (as sodium acetate). In the batch incubation experiment, tanks 1, 2 and 3 were operated separately with different concentrations of COD, which resulted in the [COD]/[NH_4_^+^] ratios of 1.4, 1.1 and 0.7 in the influents respectively. Within 30 days of the experiment, water samples from the tanks were collected every 24 h to monitor changes in the COD and inorganic nitrogen species (NH_4_^+^, NO_2_^−^, NO_3_^−^ and N_2_), as well as the redox potential (RP). The [COD]/[NH_4_^+^] ratio giving the best NH_4_^+^ removal rate was chosen for the experiment conducted under a continuous operating mode.

### Experiment of co-removal of NH_4_^+^ and COD in the continuous operating mode

Co-removal of NH_4_^+^ and COD was demonstrated in the laboratory-scaled system operated under continuous mode using an anoxic influent containing 50 mg L^−1^ NH_4_^+^ and COD (as loaded sodium acetate) at the concentration that enabled the best NH_4_^+^ removal rate. The influent was pumped into the system at a hydraulic loading rate of 2 mL min^−1^ (3 L day^−1^). Accordingly, the retention times of the influents in the three sequential tanks 1, 2, and 3 were 2, 4 and 6 days, respectively. Water samples were collected every 24 h from each tank to quantify NH_4_^+^ and COD concentrations.

### Fluorescence in situ hybridization

Precipitates scratched from HDPE MBBR matrix surfaces were fixed for 12 h at 4 °C in 4% formaldehyde, washed twice with PBS (sodium phosphate 10 mM, pH 7; NaCl 130 mM), and stored in PBS:ethanol (1:1) solution at − 20 °C. Prior to hybridization, the fixed precipitates were collected on polycarbonate filters (0.2 μm pores, Millipore). The filters were then cut into small sections, hybridized with Cy3-labelled GAM42a and Delta385 16S rRNA probes specific for γ- and δ-proteobacteria, respectively at appropriate stringencies (formamide concentrations)^15^. DAPI (4′, 6-diamidino-2phenylindole) was used for general cell staining. The hybridized samples were then observed by a fluorescence microscope (Carl Zeiss, Germany) and the fluorescence signals were captured by an AxioCam ICc3 camera. The images were then analyzed by Image J software.

### Analytical methods

The ammonium concentration was determined photometrically by using sodium nitroprusside reagent^16^. The calibration curve of the NH_4_^+^ concentration was obtained from a serial dilution of the NH_4_Cl solution (using previously dried NH_4_Cl crystals) in the range of 0.018–1.8 mg L^−1^.

The ferrous iron concentration was determined photometrically by using O-phenanthrolin reagent^17^. A calibration curve was obtained from a serial dilution of the FeSO_4_ solution in the range of 0.28–2.8 mg L^−1^.

The nitrate concentration was determined by using NitraVer^®^ 5 Nitrate Reagent Powder Pillows (Hach Instruments Inc., USA) with a calibration curve obtained from a serial dilution of the NaNO_3_ solution in the range of 0–30 mg L^−1^.

The nitrite concentration was determined by using NitriVer^®^ 3 Nitrite Reagent Powder Pillows (Hach Instruments Inc., USA) with a calibration curve obtained from a serial dilution of the NaNO_2_ solution in the range of 0–150 mg L^−1^.

Quantification of N_2_ was conducted by gas chromatography (GC) on 7890A instrument (Agilent) using the column HT-plot/Q with a TCD detector. The GC operating parameters were as follows: oven temperature 60 °C, detector temperature 250 °C, argon carrier gas (Messer, Vietnam) at a flow rate of 3.0 mL min^−1^, and 0.2 mL sample injection volume. An N_2_ calibration curve was constructed by using a mixture of N_2_ in argon in the range 0–100% volume prepared in serum bottles tightly closed with rubber stoppers. The concentration of N_2_ was calculated as % in samples based on the width of the peak values.

COD determination was conducted according to USEPA 410.4 method by using COD digestion vials with 1000 mg L^−1^ COD standard solution (Hach Instruments Inc., USA).

The redox potential (RP) of the medium in the treatment system was measured by using a working graphite electrode in close proximity with an Ag/AgCl reference electrode (BASi, West Lafayette, IN 47906, USA), with a digital multimeter (model 1009, Kyoritsu, Japan). Both electrodes were built on a rubber stopper of a 100 mL Schott flask. Thus, 80 mL liquid sample was taken from the treatment system into the 100 mL Schott flask, and then the flask was immediately sealed with the rubber stopper that carried the working and reference electrodes. The RP was subsequently measured by the digital multimeter using the voltammetry mode (Fig. S3).

### Data analysis

All measurements were carried out in triplicate, unless otherwise stated. The data collected during the experiments were processed using Microsoft Excel software (for average and standard deviation functions, the standard deviation was calculated using the "n-1" method) and graphed by using SigmaPlot 14 software.

The C and N balance calculations were carried out with experimental data retrieved from the batch incubation experiment in tank 1 where the best NH_4_^+^ removal was observed (details are presented in supplementary material).

## Results

### Enrichment of iron-reducing community

**Figure 2:**
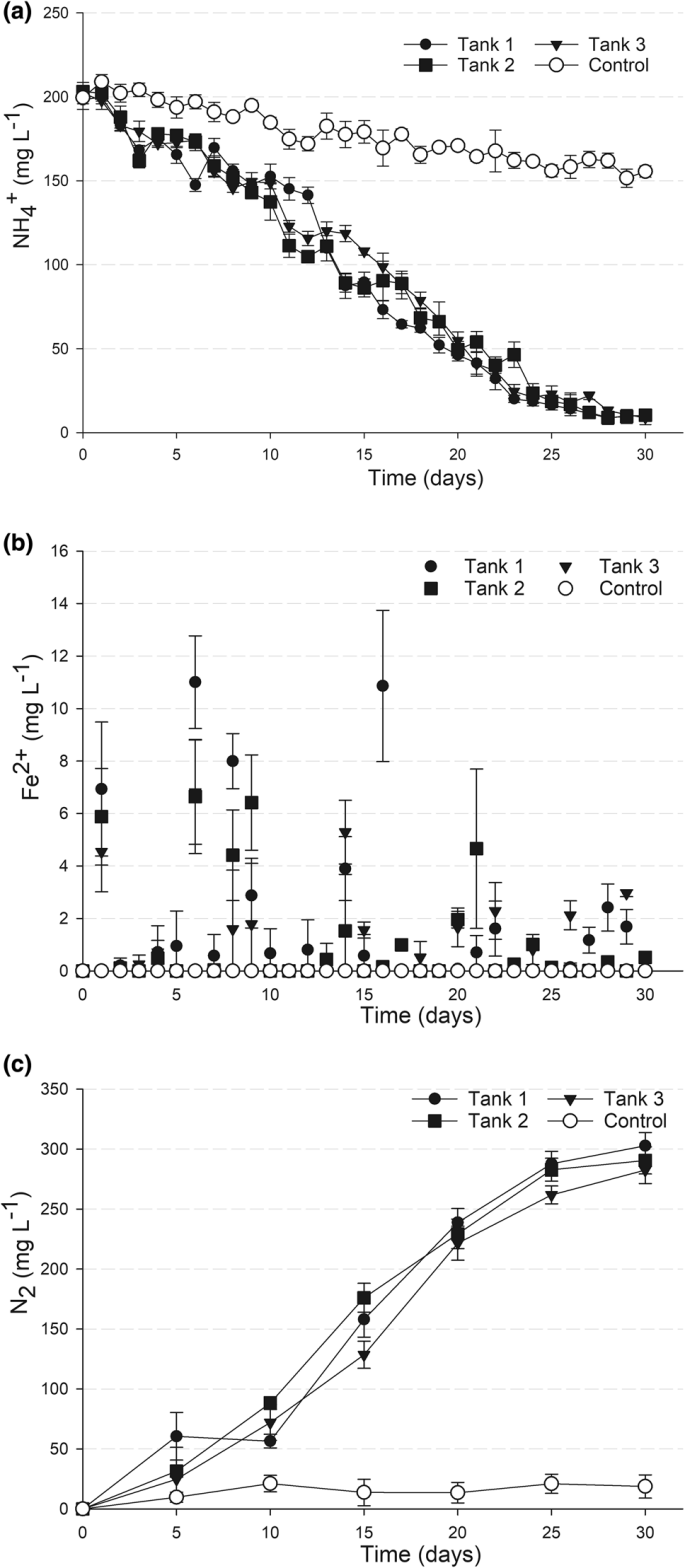
Enrichment of the feammox community in the laboratory-scaled system. The NH_4_^+^ oxidation (**a**) coupled with Fe^2+^ production (**b**) leading to N_2_ formation as product of NH_4_^+^ conversion (**c**) during the enrichment. The mineral medium used for enrichment contained 200 mg L^−1^ NH_4_^+^ and 250 mg L^−1^ COD (as sodium acetate). The inoculum was anoxic sludge from the wastewater treatment station in Cu Chi (HCM city, Vietnam). The control was a separate tank loaded with the same medium and inoculum, except for the COD content (organic carbon).

The enrichment was carried out in the laboratory-scaled treatment system with Fe-containing anaerobic sludge from a wastewater treatment plant as the initial seed. It can be seen from the results Fig. 2 that the enrichment process harbored a microbial community capable of oxidizing NH_4_^+^ and reducing Fe^3+^, as the NH_4_^+^ concentration decreased while Fe^2+^ was released into the medium. In all three tanks NH_4_^+^ oxidation started from the 3rd day of incubation and kept occurring at a relatively stable rate toward the end of enrichment. After 30 days of incubation, NH_4_^+^ was almost completely removed, from 200 mg L^−1^ to < 10 mg L^−1^ in all three tanks (Fig. 2a). In the negative control without organic carbon, NH_4_^+^ conversion was much lower, with a remained concentration as high as 155 mg L^−1^ after 30 days.

In accordance with NH_4_^+^ conversion, Fe^2+^ was detected as the product of Fe^3+^ reduction (Fig. 2b), the concentration was up to 11.01 mg L^−1^ in the medium. It could be observed that the dissolved Fe^2+^ detected in the enrichment medium was not in a cumulative manner throughout the enrichment, at certain time points its concentration was even critically low (Fig. 2b). During the enrichment process, the pH in the system was relatively stable in the range of 6.8–7.2. Distinct NH_4_^+^ oxidation in the enrichment experiment compared to that of the control (Fig. 2a) indicated that organic carbon is important for the Fe^3+^-dependent NH_4_^+^ oxidation in this system. Indeed, ~ 50% of COD was consumed after 30 days of the enrichment (Table 1), implying that the feammox process was coupled with heterotrophy.

**Table 1:** NH_4_^+^ and COD removal in the enrichment and the control without organic carbon (data were average of measurements in three tanks of the system).

Time (day)	Enrichment		Control	
	NH_4_^+^ (mg L^−1^)	COD (mg L^−1^)	NH_4_^+^ (mg L^−1^)	COD (mg L^−1^)
0	201.23 ± 2.67	250.21 ± 7.23	199.56 ± 7.02	0.00 ± 0.00
10	146.28 ± 7.87	228.98 ± 7.16	184.71 ± 3.23	0.00 ± 0.00
20	51.03 ± 3.35	175.85 ± 7.98	170.93 ± 1.8	0.00 ± 0.00
30	9.91 ± 0.86	128.30 ± 3.75	155.54 ± 3.31	0.00 ± 0.00

Regarding the products of NH_4_^+^ oxidation, neither NO_2_^−^ nor NO_3_^−^ was detected in the enrichment medium. Instead, N_2_ accumulated in the head spaces of all three tanks, with the highest concentration reaching 302.75 mg L^−1^ after 30 days of enrichment incubation (Fig. 2c).

### The effect of COD on NH_4_^+^ removal

After the enrichment process had succeeded in establishing an active “feammox” community in the treatment system (Fig. 2a), batch incubation experiments were carried out independently in the three tanks loaded with influents of different NH_4_^+^ and COD concentrations. In detail, the influents contained 200 mg L^−1^ NH_4_^+^ and COD (as sodium acetate) at different concentrations according to the [COD]/[NH_4_^+^] ratios of 1.4; 1.1 and 0.7 in tanks 1, 2, and 3, respectively.

The results showed that the NH_4_^+^ and COD removal efficiencies to a large extent depended on the ratio of these two chemical species in the influent (Table 2). Thus, after 30 days of incubation the highest NH_4_^+^ removal efficiency (98.3%) was achieved in the first tank where the [COD]/[NH_4_^+^] ratio was 1.4. That removal was significantly higher than in tanks 2 and 3 (83.3% to 85.5%) where the [COD]/[NH_4_^+^] ratios were lower, 1.1 and 0.7, respectively.

**Table 2:** Effect of COD on the NH_4_^+^removal in batch incubation experiment after 30 days.

Initial [COD]/[NH_4_^+^] ratio	NH_4_^+^ removed (%)	COD removed
1.4	98.3	58.8
1.1	83.3	63.7
0.7	85.5	57.9

**Figure 3:**
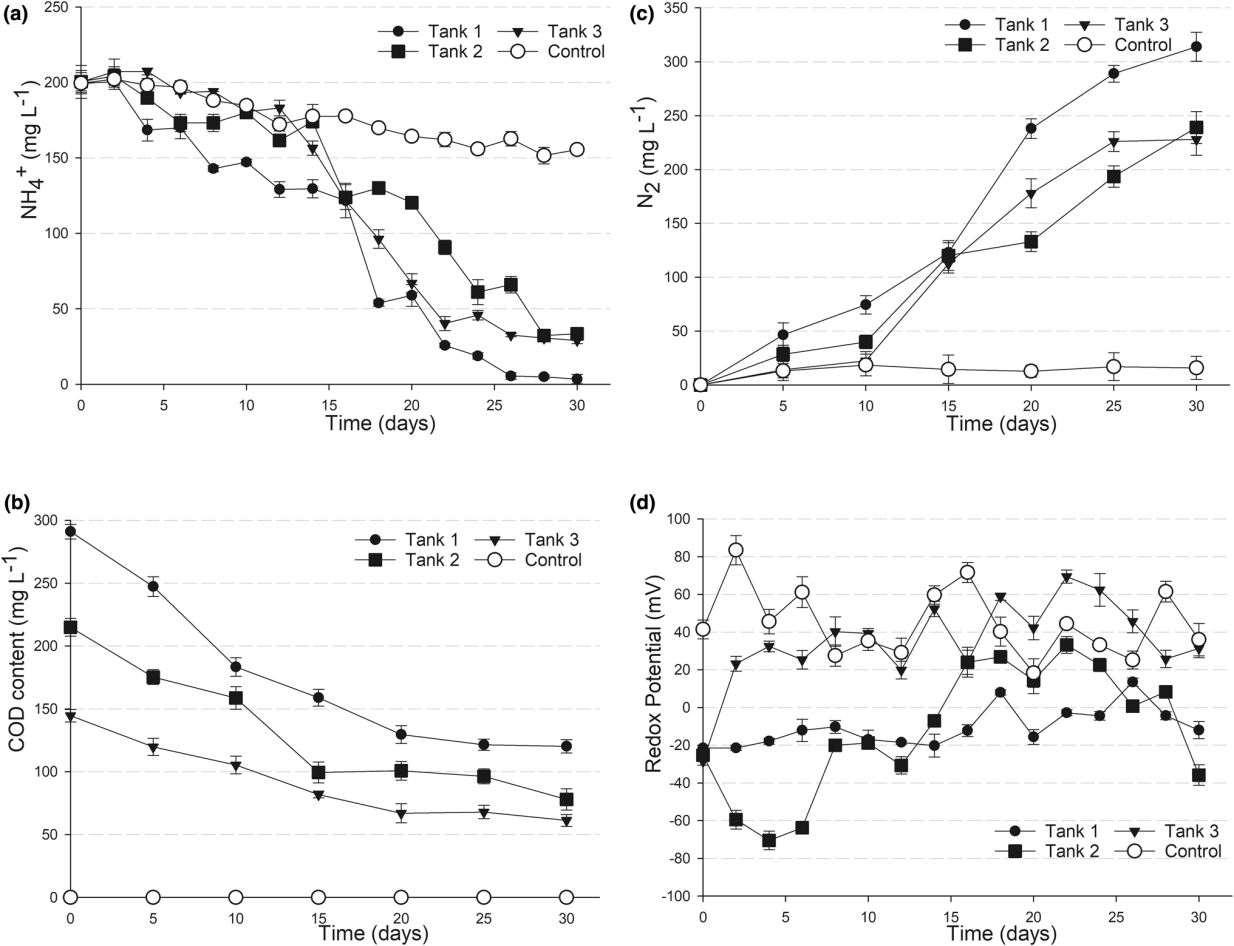
Batch incubation experiments. The removal of NH_4_^+^ (**a**) and COD (**b**); formation of N_2_ as the product of NH_4_^+^ conversion (**c**), and the redox potential (RP) of the medium (**d**) during the batch incubation with an influent containing 200 mg L^−1^ NH_4_^+^ and organic carbon (as sodium acetate) supplied in different [COD]/[NH_4_^+^] ratios: Tank 1, 1.4; Tank 2, 1.1; Tank 3, 0.7. The control was a separate tank loaded with the same influent and inoculum, except for the COD content (organic carbon).

Regarding COD removal, approximately 58% of the COD loaded was removed after 30 days in tank 1 and tank 3, where the [COD]/[NH_4_^+^] ratios were 1.4 and 0.7 respectively. Slightly higher COD removal efficiency was observed in tank 2, where the [COD]/[NH_4_^+^] ratio was 1.1 (Table 1, Fig. 3b). Thus, unlike the NH_4_^+^ removal, the COD removal was not clearly affected by the [COD]/[NH_4_^+^] ratio in the influent. Surprisingly, the COD removal varied insignificantly from 58.8 to 63.7%, despite significant variation in the [COD]/[NH_4_^+^] ratio, from 0.7 to 1.4.

Nitrate and nitrite were not detectable in all three tanks during the batch incubation experiment. Instead, N_2_ accumulated in the head space of each tank as the only product of NH_4_^+^ conversion (Fig. 3c). Gas production in all the three tanks could be observed via the formation of gas bubbles, which to different extents depended on the [COD]/[NH_4_^+^] ratio (Fig. S3). N_2_ production in tank 1 was higher than in tank 2 and tank 3, which reflects the higher NH_4_^+^ oxidation that occurred in these tanks (Fig. 3a).

The redox potential (RP) of the medium during the batch incubation experiment was also monitored to determine its correlation to the “feammox” process ( Fig. 3d). In tank 1 where [COD]/[NH_4_^+^] ratio was 1.4, the RP was most stable, varying only in the range of − 20 to + 20 mV throughout the experiment. In contrast, in tanks 2 and 3 as well as in the control without supplemented organic carbon, the RP fluctuated to a large extent, mostly in the positive ranges of + 20 mV and above. Obviously, the RP condition in tank 1 was the most suitable for the reduction of ferric iron.

Thus, via the batch incubation experiment we showed that for an influent with NH_4_^+^ concentration as high as 200 mg L^−1^ and a [COD]/[NH_4_^+^] ratio of 1.4, the NH_4_^+^ removal efficiency was impressively high, reaching 98.3%. Simultaneously, 58.8% COD was removed. This process occurred under the conditions of relatively stabilized neutral pH levels (6.8–7.2) and redox potentials (− 20 ÷  + 20 mV).

Calculations for the nitrogen balance (Table S4) showed that throughout the incubation experiment, NH_4_^+^ was thoroughly converted to N_2_ according to the stoichiometric ratio 1:0.5 (reaction 1). Acetate consumptions at days 5, 10 and 15 were in accordance with the theoretical ratio that could be used for NO_3_^−^ or Fe^3+^ reduction (reactions 4 and 5), taking into account that NO_3_^−^ might have been generated as product of feammox reactions 2 and 3.

4 $${\text{NO}}_{{3}}{^{ - }} + { 5}/{\text{8CH}}_{{3}} {\text{COO}}^{ - } + { 13}/{\text{8H}}^{ + } \to 0.{\text{5N}}_{{2}} + { 5}/{\text{4CO}}_{{2}} + { 7}/{\text{4H}}_{{2}} {\text{O }} \quad ( - {5}0{\text{1 kJ mol}}^{{ - {1}}} )$$

5 $${\text{Fe}}^{{{3} + }} + { 5}/{\text{8CH}}_{{3}} {\text{COO}}^{ - } + { 1}/{\text{2H}}_{{2}} {\text{O }} \to {\text{ Fe}}^{{{2} + }} + { 1}/{\text{4HCO}}_{{3}}{^{ - }} + { 9}/{\text{8H}}^{ + } \quad ( - {\text{814 kJ mol}}^{{ - {1}}} )$$

However, this trend was not observed at days 20, 25, and 30 later, as the amounts of oxidized acetate were lower than the theoretical values, i.e. 56% at day 20; 45.8% at day 25, and 42.5% at day 30 (Table S4). The oxidation of acetate to CH_4_ was not observed (Fig. S2B).

### Co-removal of NH_4_^+^ and COD in a continuous operation mode

**Figure 4:**
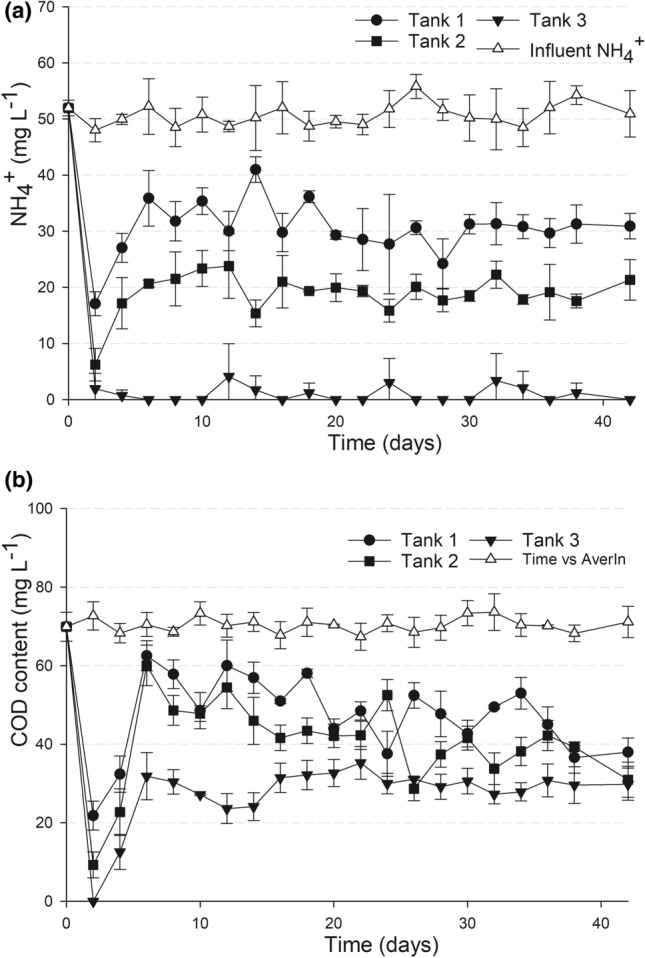
Performance of the laboratory-scaled feammox system when operated in continuous mode. The removal of NH_4_^+^ (**a**) and COD (**b**) under the feammox-coupled-with-heterotrophy condition in the system with the influent containing 50 mg L^−1^ NH_4_^+^ and [COD]/[NH_4_^+^] = 1.4. The hydraulic loading rate was 2 mL min^−1^ and the retention times in the three tanks were 2, 4 and 6 days, respectively.

Continuous operation mode in the laboratory system was carried out to assess the efficiencies of NH_4_^+^ and COD removals under the most suitable conditions, as determined in the batch experiment above. In this experiment, an influent with the moderate NH_4_^+^ concentration of 50 mg L^−1^ and [COD]/[NH_4_^+^] ratio of 1.4 was applied at the hydraulic load of 2 mL min^−1^. As a result, NH_4_^+^ was removed constantly at different levels depending on the retention time in each tank of the system (Fig. 4a). The NH_4_^+^ removal required only a short time for adaptation, readily reaching a steady state after 5 days of continuous operation, with the removal efficiency maintained at around 30%, 60% and > 91% in tank 1, tank 2, and tank 3, respectively. The most efficient NH_4_^+^ removal was achieved at 6 day retention time in tank 3, with a stabilized efficiency of > 91% (Tab. S1). As a result, an NH_4_^+^ concentration as low as 0–4 mg L^−1^ was maintained in the effluent at a retention time of 6 days (Fig. 4a, Tab. S1).

Regarding COD removal, a steady removal rate was achieved after 5 days of operation, which was consistent with the NH_4_^+^ removal (Fig. 4b). The highest level of COD removal was observed in the third tank, i.e. with a 6 day retention time and efficiency in the range of 54–66% throughout 30 days of continuous operation (Fig. 4b, Tab. S2). Accordingly, the COD concentration in the effluent was in the range of 23–30 mg L^−1^ (Tab. S2) throughout the experiment.

**Figure 5:**
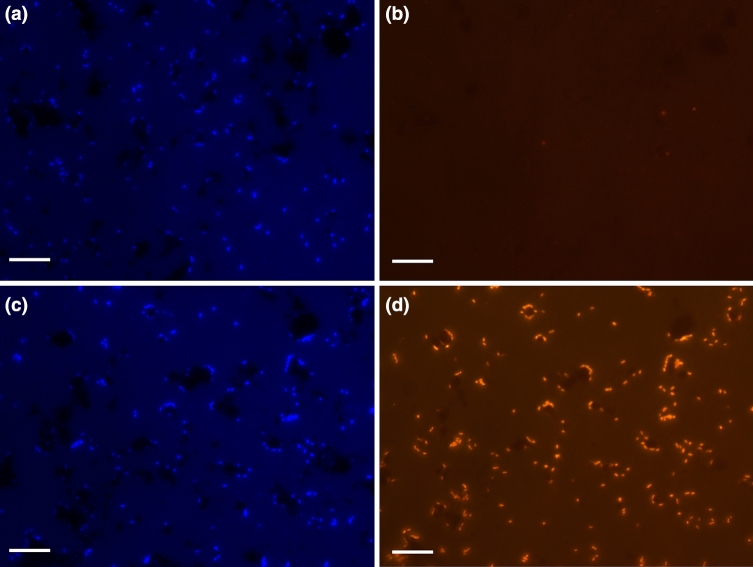
Images of cells scratched from the HDPE MBBR matrix surfaces with signals from the DAPI stain (**a**,**c**) and of the same section with signals from hybridized Cy3-labeled Delta385 and GAM42a 16S rRNA oligonucleotide probes specific for δ-proteobacteria (**b**) and γ-proteobacteria (**d**), respectively. Bar, 10 μm.

Fluorescence in situ hybridization using two probes specific for the δ- and γ-proteobateria showed that the number of DAPI-stained cells hybridized with the δ-proteobacteria specific probe was quite low (Fig. 5a,b). In contrast, a very high number of cells gave signals with the γ-proteobateria specific probe (Fig. 5c,d), which accounted for more than 90% of the total DAPI cell count (Tab. S3).

## Discussion

The NH_4_^+^ oxidation coupled with ferric iron reduction called feammox was first proposed in^2^ as an important step of the nitrogen cycle in saturated sediment^8^. Since then, the feammox process has been proven to occur in different habitats where NH_4_^+^ can be oxidized to N_2_, NO_2_^−^ or NO_3_^−^, depending on the pH levels in the environments^6^. Regarding its application in NH_4_^+^ removal from wastewater, feammox seems to be a promising new treatment technology, that is alternative to conventional nitrification/denitrification and partial nitritation/anammox processes.

The first demonstration of NH_4_^+^ removal by feammox process as a possible wastewater treatment technology was carried out in a pH and oxygen controlled laboratory fixed bed reactor^4^. The reactor was supplied with NH_4_^+^ and Fe(III)EDTA, operated in bicarbonate buffered environment with neutral to slightly alkali pH^4^. Under such conditions, NH_4_^+^ was oxidized to NO_2_^−^, thereby raising the pH from 6.7 to 7.8 (due to intensive consumption of protons, according to reaction 2). No organic carbon was added, instead, feammox microorganisms in the reactor produced organic compounds to some extent. More recently, Huang and Jaffé described the enrichment of feammox microorganisms in a batch incubation experiment carried out under acidic conditions (pH 5.5) without added organic carbon^10^. These authors reported a significant NH_4_^+^ oxidation to NO_2_^−^ when ferrihydrite was used as the only electron acceptor. Both studies showed the feammox process in lithotrophic environments with NH_4_^+^ as the only electron donor for ferric iron reduction, which led to the production of NO_2_^−^. Thus, to thoroughly convert NH_4_^+^ from the wastewater to dinitrogen gas in these studies, two more steps would be needed, i.e. (i) nitrite oxidation to nitrate, which is lithotrophic and requires oxygen and (ii) nitrate reduction to N_2_, which is heterotrophic and does not require oxygen.

In this study we showed that at a neutral pH and in the presence of organic carbon, feammox was likely to occur with a thorough NH_4_^+^ conversion to N_2_ (reaction 1). The role of organic carbon in the feammox process was evident via a significantly distinct conversion of NH_4_^+^ to N_2_ in the experimental tanks in the presence of organic carbon in comparison with that in the control without organic carbon (Fig. 2a,c). This conversion was in good agreement with the stoichiometric ratio of NH_4_^+^ to N_2_ according to reaction 1. Consumption of organic carbon was evident in all experiments, i.e. enrichment (Table 1), batch incubation (Fig. 3b) and continuous operation (Fig. 4b), indicating that the feammox process was coupled with heterotrophy. Carbon balance calculations (Table S4) also confirmed that acetate was likely to be utilized as the carbon source in the feammox process. On the other hand, Fe^3+^ reduction using acetate as the electron donor was also feasible under the experimental conditions. It is theorized that Fe^3+^ reduction with acetate might have been dominant in the system throughout the first two weeks, but was soon replaced by the feammox to N_2_ reaction, which required less acetate.

In the context of wastewater treatment, anaerobic processes are considered to be more advantageous as energy is saved. In the anaerobic world, ferric iron Fe^3+^ is a special electron acceptor that differs from others by two characteristics, i.e. (i) being water-insoluble and (ii) continuity of the two iron species Fe^3+^⇆ Fe^2+^, whereas the products of nitrate reduction N_2_, sulfate reduction SH^−^ or methanogenesis CH_4_ would escape from the liquid. Thus, in practice of wastewater treatment, if ferric iron is used as the electron acceptor, it would not need to be added regularly. In daily life, reactive iron-rich sources are relatively abundant, e.g. iron rich sludge in drinking water treatment plants or red mud from bauxite tailing^18^. From the viewpoint of achieving an eco-friendly approach, utilizing iron-rich wastes is of special consideration.

In daily life, wastewaters with high NH_4_^+^ and COD contents such as discharge from seafood processing plants, poultry farms, or biogas effluents, are very common. Such kinds of wastewater sources are considered to be highly contaminated and must be subjected to treatments to remove NH_4_^+^, COD and other contaminants before discharging to the surrounding environment. Thus, in the present study, we attempted to establish an efficient treatment system based on the feammox principle to remove NH_4_^+^ in heterotrophic conditions, i.e. in the presence of organic carbon (feammox-coupled-with-heterotrophy condition), which is common to many types of wastewater sources in daily life. The heterotrophic feammox community in this treatment system was enriched from Fe-containing sludge collected from an anaerobic wastewater treatment plant in southern Vietnam. The original treatment plant had been working under a feammox-resembling condition, i.e. with a high NH_4_^+^ concentration (~ 300 mg L^−1^) and excessive Fe^3+^ content in the form of red mud (bauxite residue), which raised the pH in the system to 7.5 ± 0.2. In addition to the high NH_4_^+^ concentration, the influent also had a high COD concentration of ~ 2500 mg L^−1^. The working conditions of our laboratory-scaled system followed the original treatment system (Fig. S1) from where the seed sludge was obtained. It should be noted that the working conditions we applied to our laboratory system were far different from those in Sawayama’s^4^ and Huang’s^10^ studies (discussed above) in several aspects, i.e. occurrence of heterotrophy, the high load of NH_4_^+^ and neutral pH in the influent. Under such conditions, parallel removals of NH_4_^+^ and organic carbon coupled with biological Fe^3+^ reduction was evident. It was of special interest that N_2_ (neither NO_2_^−^, nor NO_3_^−^) was detected as the only product of NH_4_^+^ conversion in the system. Still, it would be early to conclude that the conversion of NH_4_^+^ to N_2_ was due to the feammox process alone, since in such a complex system, other bacterial groups of nitrogen cycling might also exist.

In this study, based on the Fe^2+^ concentration detected in the medium, it is evident that it did not accumulate in the theoretically expected manner (Fig. 2a,b; Fig. S4). This might be due to the complexity of the system, in which the produced Fe^2+^ could be involved in different chemical transformations. The disappearance of Fe^2+^ when produced in the enrichment environment can be explained by two processes: (i) precipitating with carbonate as white deposition in the enrichment tanks, and/or (ii) reoxidizing to Fe^3+^ by reacting with trace oxygen in the medium or (more likely) via biological oxidation coupled with nitrate reduction^8^. Indeed, the RP in the medium during the batch incubation experiment ranged from − 20 mV to + 20 mV, which is feasible for both Fe^3+^- and NO_3_^−^-reduction processes. Thus, the collected data of the Fe^2+^time course could not be used as evidence to support the Fe^3+^ reduction process in the system.

It should be noted that the microbial community in the treatment system presented in this study did work with a very high influent NH_4_^+^ concentration of 200 mg L^−1^. Nevertheless, NH_4_^+^ was almost completely removed after 30 days of incubation (Fig. 3a). The wastewater treatment practice showed that such a high NH_4_^+^ content could not be easily removed by the conventional nitrification/denitrification process, whereas a complex operating condition and energy would be required for the partial nitritation/anammox process^19,20^.

According to Huang and Jaffé, the NH_4_^+^ removal efficiency of 9.8% was achieved in a feammox membrane reactor after 6 days of operation, and and efficiency of 64.5% was recorded for longer period of 150 days^10^. Similarly, a low NH_4_^+^ removal of 20% after 50 days was observed in the feammox reactor established by Sawayama^4^. Such a low rate of NH_4_^+^ removal was also observed in the control without added organic carbon in this study (Figs. 2a, 3a). It was surprising that the presence of COD significantly enhanced NH_4_^+^ removal, as shown by our results. However, the amount of COD needs to be controlled to achieve a certain ratio of [COD] to [NH_4_^+^] that can enable the most efficient NH_4_^+^ removal. Thus, by setting the [COD]/[NH_4_^+^] at the ratio of 1.4, almost all NH_4_^+^ was removed from such a high concentration of 200 mg L^−1^ after 30 days of batch incubation. Similarly, under a continuous operation mode, the same [COD]/[NH_4_^+^] ratio was applied to a moderately contaminated influent (containing 50 mg L^−1^ NH_4_^+^), which succeeded in more than 91% NH_4_^+^ removal with a retention time of only 6 days. At the same time, COD was efficiently removed, which resulted in an effluent with low contents of NH_4_^+^ (Tables S1 and S2).

Studies on species composition and key players involved in the NH_4_^+^ removal process under iron reduction conditions in bioreactor systems so far have led to equivocal results. In the study of the feammox process under neutrophilic autotrophic condition using Fe(III)-EDTA as the electron acceptor for the ammonium oxidation, Sawayama^4^ reported a bacterial community dominated by *Exiguobacterium* spp., *Pseudomonas* spp. and *Carnobacterium* sp. (with 22.5%, 17.5%, and 7.5% of the 16S rRNA gene clone library, respectively)^4^. Being previously reported as arsenate reducers, bacteria belonging to the *Exiguobacterium* group were believed to be candidates for the feammox process^4,21^. In the bioreactor operated under acidic autotrophic feammox conditions, a complex microbial community with the key player *Acidimicrobiaceae* bacterium A6 was identified^10^. It could be deduced that under heterotrophic feammox conditions such as in this study, the established microbial community would be quite different from that under the auto-lithotrophic conditions demonstrated by Sawayama^4^ or Huang and Jaffé^10^. The absence of δ-proteobacteria in the community established in the treatment system presented here is in agreement with the two reports mentioned above. The predominating γ-proteobacteria (more than 90% of the DAPI stained cells attached to the HDPE MBBR matrix surfaces) suggests that known iron reducers of the *Pseudomonas* and *Shewanella* genera or other unknown species would be key players^22^. It should be emphasized that the community established in this study oxidized NH_4_^+^ thoroughly to N_2_, while the other two communities reported by Sawayama^4^ and Huang and Jaffé^10^ oxidized NH_4_^+^ only to NO_2_^−^, for which further conversion steps would be needed to eliminate nitrogen from wastewater.

In conclusion, at a neutral pH, the feammox is likely to occur intensively if organic carbon was present. The highly efficient NH_4_^+^ removal process based on the feammox-coupled-with-heterotrophy principle presented in this study would have substantial application potential as a novel technology for wastewater treatment. Evidentially, applications using this principle would drive thorough NH_4_^+^ removal from wastewater while producing N_2_ as the end-product in a single-step treatment. Occurring at a neutral pH, the feammox to N_2_ coupled with heterotrophy as presented in this study would be applicable for a wide range of wastewater sources.

## Supplementary Information

Supplementary Information.
