# Supplementary material for: Ammonium and organic carbon co-removal under feammox-coupled-with-heterotrophy condition as an efficient approach for nitrogen treatment
Source: Sci Rep. 2021 Jan 12;11:784. doi: 10.1038/s41598-020-80057-y (PMC7803747; doi:10.1038/s41598-020-80057-y)
Supplement: Supplementary file 1 — Supplementary Information. [file 41598_2020_80057_MOESM1_ESM.docx]

SUPPLEMENTAL MATERIAL:

**Manuscript: AMMONIUM AND ORGANIC CARBON CO-REMOVAL UNDER FEAMMOX-COUPLED-WITH-HETEROTROPHY CONDITION AS AN EFFICIENT APPROACH FOR NITROGEN TREATMENT**

Chung Phuong Le^1,4^, Hai Thi Nguyen^2^, Toi Duy Nguyen^2^, Quyen Huynh Minh Nguyen^2^, Hai The Pham^3,4^ and Hang Thuy Dinh^2,^*

^1^Nha Trang University, Nguyen Dinh Chieu 2, Nhatrang, Khanhhoa, Vietnam

^2^VNU-Institute of Microbiology and Biotechnology, Xuan Thuy 144, Caugiay, Hanoi, Vietnam

^3^GREENLAB - Center for Life Science Research (CELIFE) and ^4^Department of Microbiology, Faculty of Biology, VNU University of Science, Nguyen Trai 334, Thanh Xuan, Hanoi, Vietnam

* Corresponding author:

Hang T. Dinh; VNU Institute of Microbiology and Biotechnology

Mailing address: E2 Building, Xuan Thuy 144, Caugiay, Hanoi, Vietnam;

phone: +84 (0) 972 523 466; fax: +84 (0) 2437547407

e-mail: [dthangimbt@gmail.com](mailto:dthangimbt@gmail.com); webpage: <http://imbt.vnu.edu.vn>

**Supplemental methods**

- 1. **Microbial inoculation for the enrichment of feammox microorganisms**

The seed sludge used in the enrichment experiment was collected from anaerobic tank of wastewater treatment station in Cu Chi district, HCM City, Vietnam (Fig. S1)


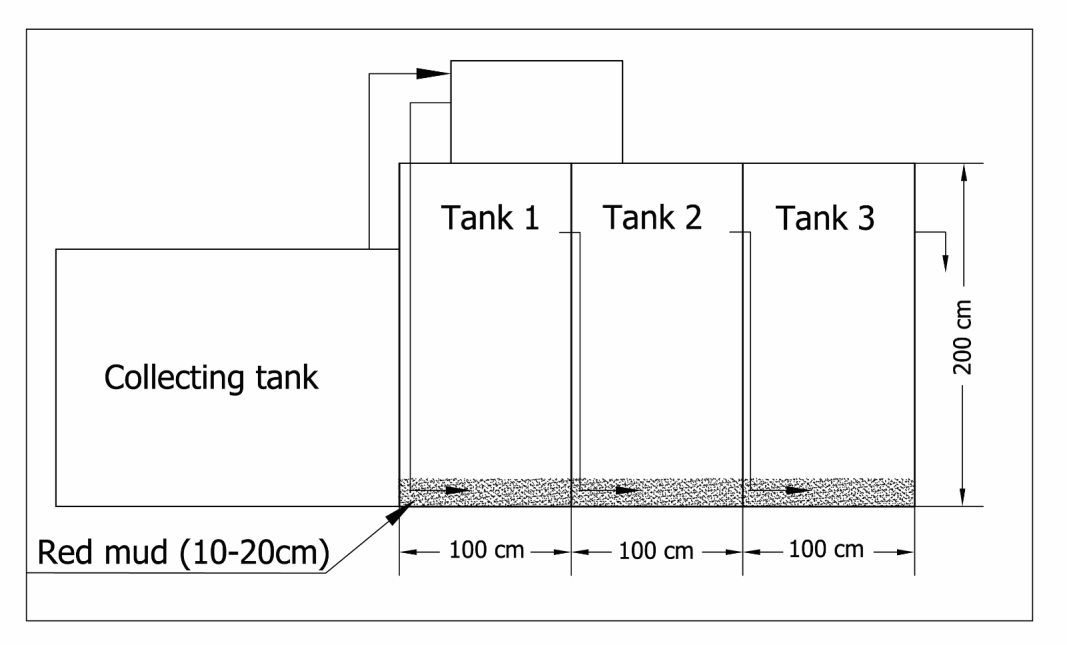


**Figure S1.** A diagram showing operation principle of the wastewater treatment station in Cu Chi district, HCM City, Vietnam where the anaerobic sludge was taken for the use as seeding in the enrichment of feammox microorganisms in this study. Arrows show moving direction of wastewater inside the treatment system.

The red mud used at the treatment plant contained 45% iron of two forms goethite (FeOOH) and hematite (Fe_2_O_3_), the total Fe^2+^ concentration was ~2.0 mol L^−1^ (personal communication with Dr. Hoang Dong Nam, who was in charge in controlling the plant). The red mud layer in each tank was 10 – 20 cm thick, slightly mixed by the movement of wastewater streams through bottom oriented tubes. The plant ran with a heavily polluted wastewater from aquaculture processing factory, i.e. contained NH_4_^+^ 300 – 350 mg L^−1^ and COD at 500 – 2500 mg L^−1^.

- 1. **Preparation of ferrihydrite slurry^1^**

Ferrihydrite slurry was prepared according to Ratering (1999). In brief, 1,000 mL of 0.25 M FeCl_3_.6H_2_O solution was added to a 5,000 mL glass ﬂask with 740 mL of 1 M NaOH solution, vigorously stirred to raise pH of the mixture to 7.0. Afterward, 1M NaOH solution was added dropwise until the pH reached 7.6. The mixture was then centrifuged for 10 minutes at 10,000 rpm and the supernatant was removed to collect the newly formed ferrihydrite precipitates. Wash the ferrihydrite precipitates in distilled water for three times to get rid of exceeded chlorine ion (Cl^−^). After the last washing, the precipitates were mixed in 200 ml MQ and ferrihydrite content in the suspension was determined by taking 5 ml into a petri dish, drying and then weighing (performed in triplicates). The ferrihydrite prepared by this procedure did not contain any nitrate or other nitrogen species.

- 1. **Determining ammonium concentration**

Ammonium concentration was determined by using sodium nitroprusside reagent (DIN 38406-5, 1983)^2^. The sodium nitroprusside solution (solution A) was prepared by dissolving 3 g phenol and 0.003 g Na-nitroprusside in 100 mL distilled water. An alkali solution (solution B) containing 2 g NaOH and 2 mL of 13% NaClO in sufficient distilled water to a volume of 100 mL was freshly prepared prior to the experiment and stored at 4 °**C**.

Water samples of a volume of 0.02 mL were taken into test tubes, each contained 4.98 mL H_2_O (dilution rate of 1:250). Stepwise, 0.5 mL of solution A and 0.5 mL of solution B were added and mixed well, then the reagent tubes were closed and kept at room temperature (25 ± 3 ^o^C) for 1 hour in the dark. Absorbance of the samples was measured at 635 nm against pure water reference.

- 1. **Determining ferrous iron concentration**

Ferrous iron concentration was determined by using O-phenanthrolin reagent *(*DIN 38406 E1-1, 1983)^3^. Wastewater samples were collected and the pH value was decreased to 1.0 with H_2_SO_4_ solution (1% volume). To 50 mL sample added 5 mL of 5.2 M ammonium acetate and 2 mL of 1.4 M hydroxyl ammonium chloride, mixed well by vortexing (the pH of mixture should be between 3.4 – 5.5, optimal at 4.5). Afterward, 2 mL of 21 mM phenanthroline solution was added, mixed well and filled with water to 100 mL, mixed again then the tubes were kept at room temperature for 15 minutes. Absorbance of the samples was measured at 510 nm against pure water reference.

- 1. **Determining redox potential of the influents**


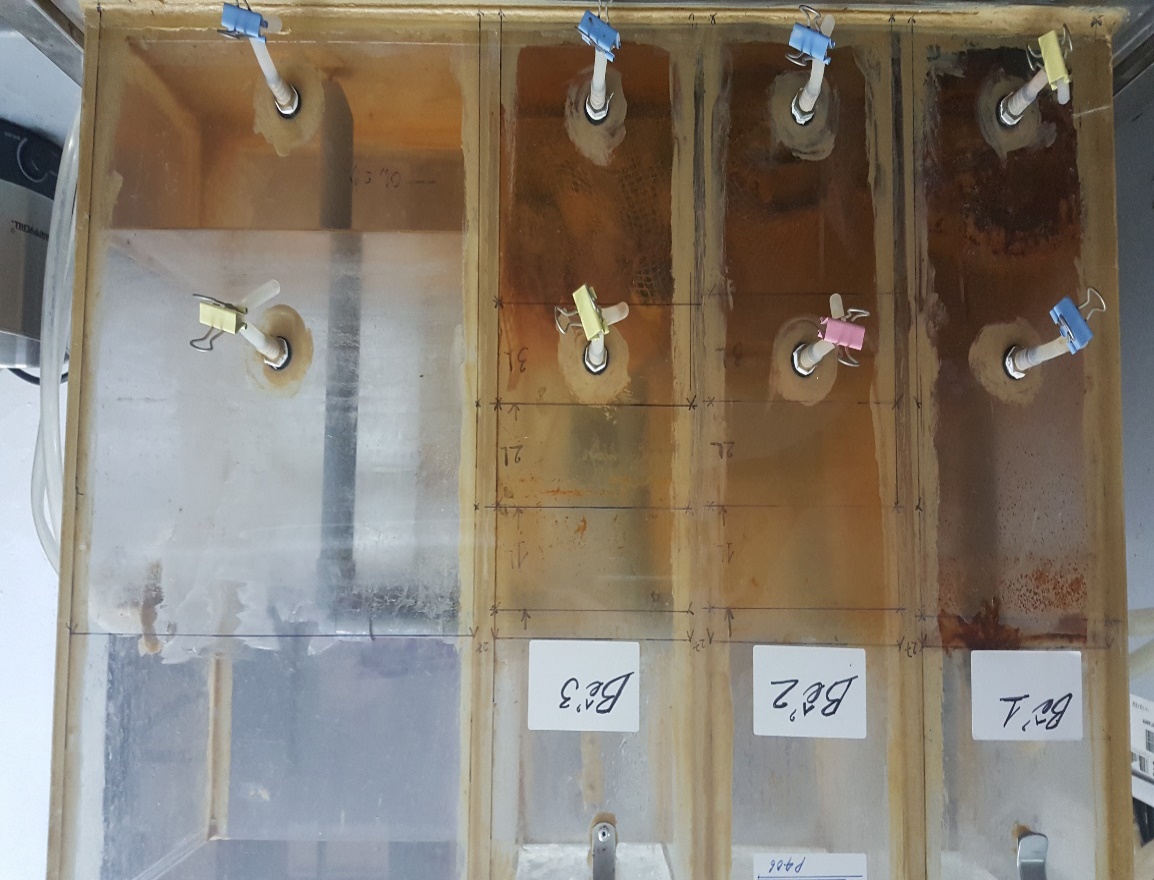
 **
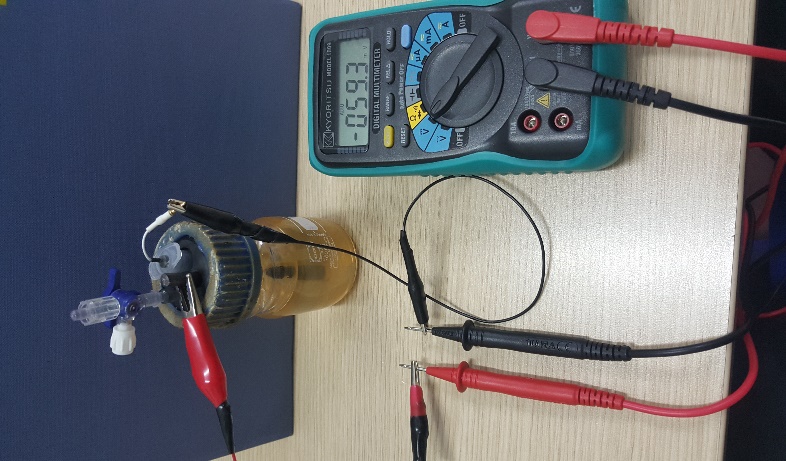
**

Tank 3

Tank 2

Tank 1

**Figure S3.** A diagram showing procedure of taking water samples and measuring the redox potential of the influents by voltmeter

- 1. **Vitamin Mixture^2^**

| Na_2_HPO_4_/NaH_2_PO_4_ 25 mM, pH 7.1 | 100 mL |
| --- | --- |
| 4-Aminobenzoic acid | 4 mg |
| D(+)- Biotin | 1 mg |
| Nicotinic acid | 10 mg |
| Calcium D(+)-Pantothenate | 5 mg |
| Pyridoxin dihydrochloride | 15 mg |
| Lipoic acid | 1.5 mg |
| Folic acid | 4 mg |
| Na-2- Mercaptoethane sulfonate | 25 mg |
| The solution was filter-sterilized (pore size, 0.2 µm) and kept in the dark at 4 ^o^C. | |

- 1. **Trace Element Mixture^2^**

| Na_2_EDTA | 5200 mg |
| --- | --- |
| FeSO_4_.7H_2_O | 200 mg |
| H_3_BO_3_ | 30 mg |
| MnCl_2_.4H_2_O | 100 mg |
| CoCl_2_.6H_2_O | 190 mg |
| NiCl_2_.6H_2_O | 24 mg |
| CuCl_2_.2H_2_O | 2 mg |
| ZnSO_4_.7H_2_O | 144 mg |
| Na_2_MoO_4_.2H_2_O | 36 mg |
| Add distilled water to 1000 mL | |

1. **Supplemental results**

**2.1. Time course of Fe^2+^ in the laboratory-scaled system under batch incubation**

The measurements of Fe^2+^ concentration were carried out daily with water samples in three tanks of the laboratory-scaled system during the batch incubation. The results (Figure S4) showed that Fe^2+^ indeed produced, i.e. the Fe^3+^ reduction has taken place. However the Fe^2+^ concentration in all three tanks fluctuated to a large extent instead of being in cumulative manner as expected. In fact, the whole amount of produced Fe^2+^ could not be recovered since a fraction might absorb on the anaerobic sludge^3^. Therefore, the collected data on Fe^2+^ could not be employed for characterizing the process in the system.


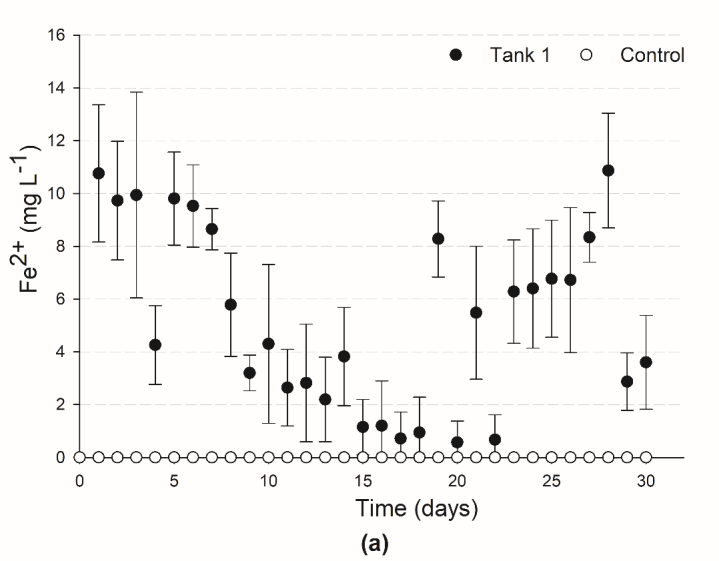


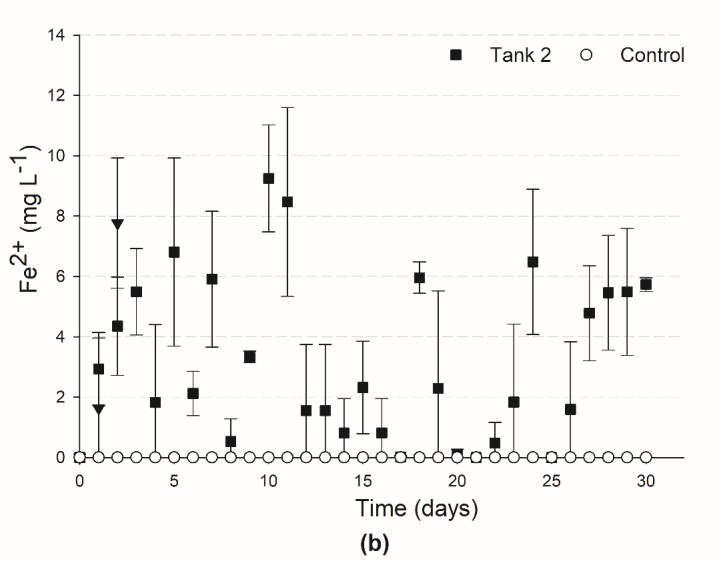


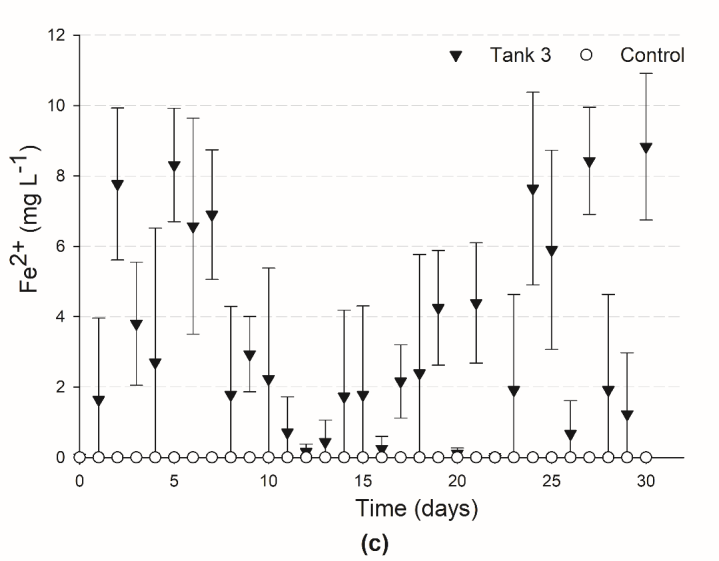


**Figure S4.** Fe^2+^ production during batch incubaction experiments. The influent containing 200 mg L^−1^ NH_4_^+^ and organic carbon supplied at different [COD]/[NH_4_^+^] ratios: Tank 1, 1.4 **(a)**; Tank 2, 1.1 **(b)**; Tank 3, 0.7 **(c)**. Control was a separate tank loaded with the same influent and inoculum, except the COD content.

**2.2. NH_4_^+^ and COD removal in the laboratory-scaled system under continuous operation**

The concentration of NH_4_^+^ and COD was measured in the effluent from Tank 3 of the treatment system periodically at 6 day intervals according to the retention time for the most efficient NH_4_^+^ removal (Table S1 and S2). The obtain values were the averages of 3 parallel measurements.

**Table S1:** NH_4_^+^ removal in the laboratory-scaled system while operating continuously

| **Time**  (days) | **Influent [NH_4_^+^]**  (mg L^−1^) | **Effluent [NH_4_^+^]**  (mg L^−1^) | **NH_4_^+^ removal** **efficiency** (%) |
| --- | --- | --- | --- |
| 0 | 51.98 ± 1.41 | 51.98 ± 1.41 | 0.00 |
| 6 | 52.25 ± 4.96 | 0.73 ± 1.03 | 98.60 |
| 12 | 48.67 ± 0.93 | 4.13 ± 5.85 | 91.51 |
| 18 | 48.72 ± 2.67 | 1.21 ± 1.71 | 97.52 |
| 24 | 51.79 ± 3.28 | 3.04 ± 4.28 | 94.13 |
| 30 | 50.19 ± 4.11 | 0.00 ± 0.00 | 100.00 |
| 36 | 52.03 ± 4.65 | 0.00 ± 0.00 | 100.00 |
| 42 | 50.93 ± 4.13 | 0.00 ± 0.00 | 100.00 |

The efficiency of NH_4_^+^ removal (%) was calculated as following:

The efficiency of NH_4_^+^ removal (%) = $\frac{\text{(}\text{Influent }\text{}\mathrm{NH}_{4}^{+}\text{}\text{) }-\text{ (}\text{Effluent }\mathrm{NH}_{4}^{+}\text{}\text{) }}{\text{(}\text{Influent }\text{}\mathrm{NH}_{4}^{+}\text{}\text{) }}$ $\times100$

**Table S2:** COD removal in the laboratory-scaled system while operating continuously

| **Time**  (days) | **Influent [COD]**  (mg L^−1^) | **Effluent [COD]**  (mg L^−1^) | **COD removal efficiency** (%) |
| --- | --- | --- | --- |
| 0 | 69.90 ± 3.68 | 69.90 ± 3.68 | 0.00 |
| 6 | 70.45 ± 3.04 | 12.55 ± 4.46 | 82.19 |
| 12 | 70.15 ± 2.90 | 23.60 ± 3.82 | 66.36 |
| 18 | 71.05 ± 3.61 | 32.15 ± 3.75 | 54.75 |
| 24 | 70.08 ± 2.12 | 29.95 ± 2.62 | 57.26 |
| 30 | 73.35 ± 3.18 | 30.06 ± 3.25 | 59.02 |
| 36 | 70.10 ± 0.29 | 30.75 ± 4.17 | 56.13 |
| 42 | 71.10 ± 3.96 | 29.80 ± 4.10 | 58.09 |

The efficiency of COD removal (%) was calculated as following:

The efficiency of COD removal (%) = $\frac{\text{(}\text{Influent }\text{}\mathrm{COD})\text{ }-\text{ (}\text{Effluent }\text{}\mathrm{COD}\text{) }}{\text{(}\text{Influent }\text{}\mathrm{COD}\text{) }}$ $\times100$

- 1. **Determining N_2_ as the product of NH_4_^+^ conversion in the batch incubations**

Gas bubbles were intensively formed in all three tanks of the system (Fig. S2A). The intensity of gas formation was most strong in Tank 1 and gradually decreased toward Tank 3 in accordance to the decrease of the [COD]/[NH_4_^+^] ratios in these tanks. Gas samples from head space of the three tanks were collected and analyzed by GC for nitrogen and other gas components. The results showed that nitrogen was the main gas in head space of all three tanks, no CH_4_ nor SH^−^ was detected (Fig. S2B).


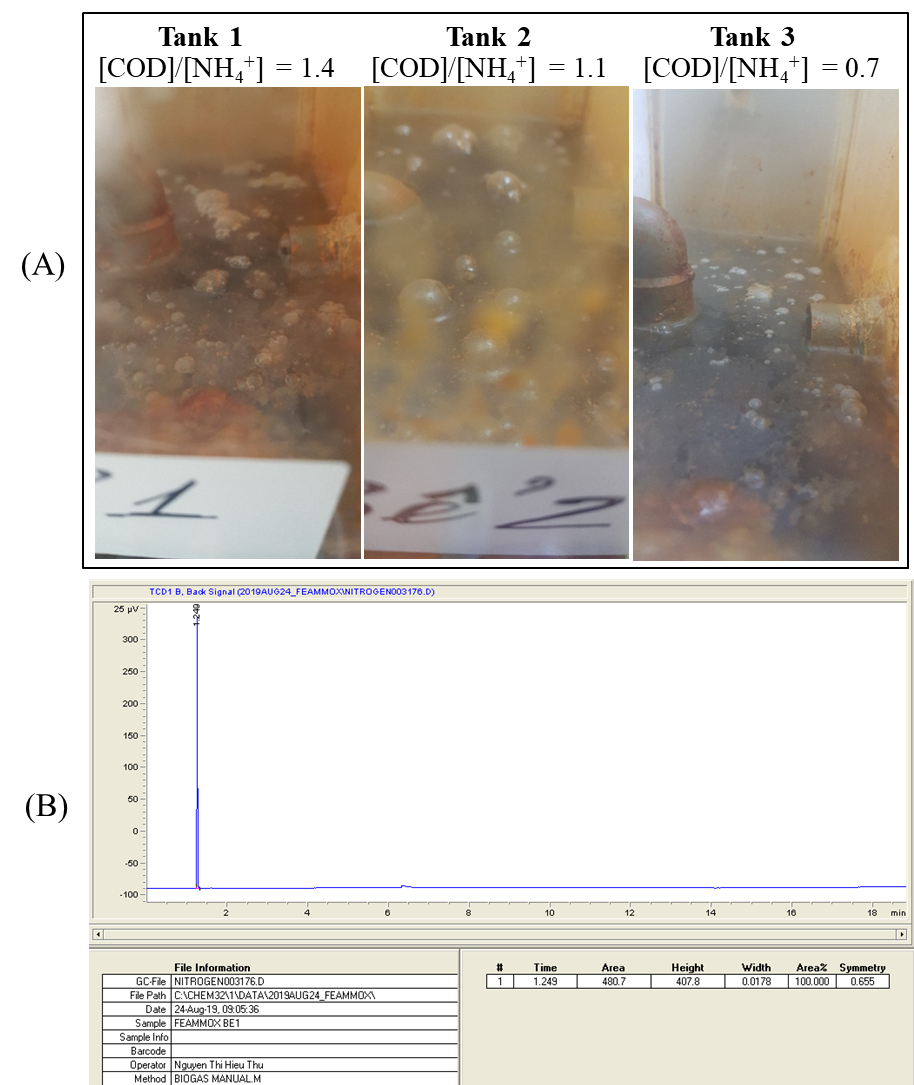


**Figure S2.** Gas bubbles observed in incubation tanks of the batch experiments (A) and chromatogram of a gas sample from Tank 1 taken at the 20^th^ day of the batch incubation experiment (B).

The N_2_ concentration (mg L^−1^ ) was calculated as following:

[N_2_] = $\frac{\text{(\% Nitrogen gas) }\text{}\text{ }V_{i}\text{ }\text{}\text{ 28}\text{ }\text{}\text{ 1000 }}{\text{22.4 }}$

Where:

(% Nitrogen gas) = percentage of N_2_ in gas sample, determined by GC.

28 × 1000 = molecular weight of N_2_ at Standard Temperature and Pressure (mg M^−1^).

22.4 = volume of 1 mole of N_2_ at Standard Temperature and Pressure (L M^−1^).

$V_{i}$ = head space volume of the tanks (Tank 1, V_1_ = 2.73 L; Tank 2, V_2_ = 2.86 L; Tank 3, V_3_ = 2.99 L).

**2.4. Counting cells in FISH experiment**

The Delta385- and GAM42a-hybridized cells on polycarbonate filters were observed under fluorescence microscope using the 40× objective. For each probes, cell numbers from ten microscopically observed fields were recorded for calculating the percentage of hybridized cells out of the total DAPI counts (Table S3).

**Table S3:** Cells counting with signals from DAPI stain and hybridization with Cy3-labelled probes in FISH experiments

| **Microscopically observed field** | **Cell counts of DAPI stain and hybridization with Cy3-labeled 16S rRNA oligonucleotide probes specific for** δ- and γ-*proteobacteria* | | | | | |
| --- | --- | --- | --- | --- | --- | --- |
|  | Delta385 (δ-*proteobacteria*) | | | GAM42a (γ-*proteobacteria*) | | |
|  | DAPI | Delta385 | % | DAPI | GAM42a | % |
| 1 | 162 | 7 | 4.12 | 205 | 196 | 95.75 |
| 2 | 104 | 3 | 2.64 | 79 | 78 | 98.11 |
| 3 | 72 | 3 | 4.61 | 37 | 33 | 90.05 |
| 4 | 80 | 8 | 9.66 | 80 | 77 | 96.07 |
| 5 | 145 | 5 | 3.45 | 187 | 176 | 94.12 |
| 6 | 138 | 6 | 4.35 | 192 | 178 | 92.71 |
| 7 | 105 | 4 | 3.81 | 89 | 82 | 92.13 |
| 8 | 91 | 5 | 5.49 | 109 | 101 | 92.66 |
| 9 | 135 | 4 | 2.96 | 181 | 168 | 92.82 |
| 10 | 56 | 2 | 3.57 | 79 | 73 | 92.41 |

**2.5. Calculation of carbon and nitrogen balance**

In the feammox process, the conversion of NH_4_^+^ would take place in different directions depending on the pH condition in surrounding environment, consequently leading to production of N_2_ at a wider range of pH (reaction 1), and to production of NO_2_^−^ or NO_3_^−^ at pH < 6.5 (reaction 2 and 3) ^4-6^.

3Fe(OH)_3_ + 5H^+^ + NH_4_^+^ → 3Fe^2+^ + 9H_2_O + 0.5N_2_ (ΔG^0^’ = −245 kJ mol^−1^) (1)

6Fe(OH)_3_ + 10H^+^ + NH_4_^+^ → 6Fe^2+^ + 16H_2_O + NO_2_^–^ (ΔG^0^’ = −164 kJ mol^−1^) (2)

6Fe(OH)_3_ + 8H^+^ + NH_4_^+^ → 6Fe^2+^ + 15H_2_O + NO_3_^–^ (ΔG^0^’ = −207 kJ mol^−1^) (3)

The stoichiometric balance of the oxidized NH_4_^+^ and the nitrogen products in the feammox processes are as following: 1 mmol NH_4_^+^ to 0.5 mmol N_2_ (reaction 1) and 1 mmol NH_4_^+^ to 1 mmol NO_2_^−^ or NO_3_^−^ (reactions 2 and 3).

For N and C balance calculation, we take data from the batch incubation experiment of Tank 1 where the [COD]/[NH_4_^+^] ratio was 1.4 and the best NH_4_^+^ removal was observed (Table S4).

**Table S4:** Chemical species of N and C conversion processes in the laboratory-scaled system

| **Time (d)** | **Oxidized reactants (mmol)** | | | **Generated products (mmol)** | | | |
| --- | --- | --- | --- | --- | --- | --- | --- |
|  | **NH_4_^+^** | **CH_3_COO^−^** | | **N_2_** | | **NO_3_^−^** | |
|  | Measured | Measured | Theory^a^ | Measured | Theory^b^ | Measured | Theory^c^ |
| 5 | 9.79 ± 1.65 | 4.44 ± 0.80 | 6.12 | 4.53 ± 1.10 | 4.90 | 0.00 ± 0.00 | 9.79 |
| 10 | 17.39 ± 0.62 | 10.96 ± 0.75 | 10.87 | 7.26 ± 0.83 | 8.69 | 0.00 ± 0.00 | 17.39 |
| 15 | 23.23 ± 1.99 | 13.44 ± 0.67 | 14.52 | 11.99 ± 0.90 | 11.62 | 0.00 ± 0.00 | 23.23 |
| 20 | 46.80 ± 2.41 | 16.42 ± 0.72 | 29.25 | 23.21 ± 0.90 | 23.40 | 0.00 ± 0.00 | 46.80 |
| 25 | 60.19 ± 0.69 | 17.25 ± 0.45 | 37.62 | 28.18 ± 0.76 | 30.10 | 0.00 ± 0.00 | 60.19 |
| 30 | 65.31 ± 1.03 | 17.37 ± 0.55 | 40.82 | 30.62 ± 1.31 | 32.65 | 0.00 ± 0.00 | 65.31 |

**^a^**The amount of acetate theoretically consumed for the reduction of NO_3_^−^ if the latter was produced in the feammox process (reaction 2 and 3) according to reaction 4: NO_3_^−^ + 5/8CH_3_COO^−^ + 13/8H^+^ → 0.5N_2_ + 5/4CO_2_ + 7/4H_2_O (ΔG^0^’ = −501 kJ mol^−1^) (4). The stoichiometric balance of NH_4_^+^ : NO_3_^−^ : CH_3_COO^−^ is 1:1:5/8.

**^b^**The amount of N_2_ theoretically produced from the oxidation of NH_4_^+^ according to reaction 1. The stoichiometric balance of NH_4_^+^ : N_2_ is 1:1.

**^c^**The amount of NO_3_^−^ can be produced in the feammox process following reaction 3 (or via NO_2_^−^ following reaction 2).

Several important points can be noted from the data presented in table S4:

- The amounts of oxidized NH_4_^+^ (mmol) and produced N_2_ were in agreement with the theoretical ratio of 1:0.5 (reaction 1), whereas no NO_3_^−^ was detected, i.e. NH_4_^+^ was thoroughly converted to N_2_.
- The COD measurements at day 5, 10 and 15 showed that the amounts of oxidized acetate were in agreement with the theoretical values required for NO_3_^−^ reduction (stoichiometric ratio 5/8 mmol acetate to 1 mmol NO_3_^−^, reaction 4), if the latter had been produced (reaction 2 and 3). However, this trend was not observed in the days after, the amounts of oxidized acetate were only accounted for 56% at day 20; 45,8 % at day 25 and 42,5% at day 30. Thus, acetate was not consumed as the electron donor for the NO_3_^−^ reduction.
- Other feasible acetate utilizing process was Fe^3+^ reduction, in which acetate can be used (i) as electron donor (in the conventional dissimilatory Fe^3+^ reduction, reaction 5) or (ii) as carbon source (in the feammox reaction generating N_2_, reaction 1). It is theorized that the Fe^3+^ reduction with acetate could dominate in the first two weeks and became soon replaced by the feammox reaction, which required less acetate.

Fe^3+^ + 5/8CH_3_COO^−^ + 1/2H_2_O → Fe^2+^ + 1/4HCO_3_^−^ + 9/8H^+^ (ΔG^0^’ = −814 kJ mol^−1^) (5)

**2.6. Enumeration of nitrate reducers in the laboratory-scaled system**

The MPN counting was carried out for denitrifiers presenting in the feammox sludge of the laboratory system. A sludge sample from the batch culture in Tank 1 was taken into a falcon tube, vigorously shaken to liberate the cells and 1 ml was used to perform serial dilution in gas tight tubes with anoxic PBS 1×. Aliquots of 1 ml from the dilution tubes were then injected into MPN serial (gas tight) tubes containing mineral medium with acetate (10 mM) as electron donor and NO_3_^−^ (5 mM) as electron acceptor. The tubes were then incubated at 30°C for 10 days in the dark. Activity of denitrifiers in the MPN tubes was determined via measuring NO_3_^−^ concentration by using NitraVer® 5 Nitrate Reagent Powder Pillows (Hach Instruments Inc., USA).

The results showed that denitrifiers presented at 10^5^ MPN ml^−1^ in the feammox sludge. Linking with the results of FISH analyses that showed γ-proteobacteria counted for more than 90% of DAPI stained cells of the feammox community in the studied system, it is plausible that the number of denitrifiers detected can be at the same time iron reducers, since genera within the γ-proteobacteria subclass such as *Pseudomonas* spp. and *Shewanella* spp. can also thrive by nitrate reduction.

1. **References for the supplement information**

1. Ratering, S. *Iron cycle in Italian rice field soil: Localization of the redox processes and characterization of the involved microorganisms.* PhD thesis, Faculty Biology, University of Marburf thesis, (1999).

2. Widdel, F. & Bak, F. in *The Prokaryotes* Vol. IV *Gram-negative mesophilic sulfate-reducing bacteria* (eds Balows, A. *et al.*) Ch. 183, 3352-3378 (Springer-Verlag, 1992).

3. Toro, E. E. R. *et al.* Anaerobic ammonium oxidation linked to sulfate and ferric iron reduction fuels nitrogen loss in marine sediments. *Biodegradation* **29**, 429-442, doi:<https://doi.org/10.1007/s10532-018-9839-8> (2018).

4. Sawayama, S. Possibility of anoxic ferric ammonium oxidation. *J. Biosci. Bioeng.* **101**, 70-72 (2006).

5. Yang, W. H., Weber, K. A. & Silver, W. L. Nitrogen loss from soil through anaerobic ammonium oxidation coupled to iron reduction. *Nature Geoscience* **5**, 538-541 (2012).

6. Huang, S. & Jaffé, P. R. Characterization of incubation experiments and development of an enrichment culture capable of ammonium oxidation under iron-reducing conditions. *Biogeosciences* **12**, 769-779 (2015).
